# An ultralow power wearable vital sign sensor using an electromagnetically reactive near field

**DOI:** 10.1002/btm2.10502

**Published:** 2023-02-26

**Authors:** Seoktae Seo, Hyunkyeong Jo, Jungho Kim, Bonyoung Lee, Franklin Bien

**Affiliations:** ^1^ Department of Electrical Engineering Ulsan National Institute of Science and Technology Ulsan Republic of Korea

**Keywords:** bioelectronics, biosensor, reactive near field, ultralow power consumption, vital sign sensor, wearable sensor

## Abstract

Despite coronavirus disease 2019, cardiovascular disease, the leading cause of global death, requires timely detection and treatment for a high survival rate, underscoring the 24 h monitoring of vital signs. Therefore, telehealth using wearable devices with vital sign sensors is not only a fundamental response against the pandemic but a solution to provide prompt healthcare for the patients in remote sites. Former technologies which measured a couple of vital signs had features that disturbed practical applications to wearable devices, such as heavy power consumption. Here, we suggest an ultralow power (100 μW) sensor that collects all cardiopulmonary vital signs, including blood pressure, heart rate, and the respiration signal. The small and lightweight (2 g) sensor designed to be easily embedded in the flexible wristband generates an electromagnetically reactive near field to monitor the contraction and relaxation of the radial artery. The proposed ultralow power sensor measuring noninvasively continuous and accurate cardiopulmonary vital signs at once will be one of the most promising sensors for wearable devices to bring telehealth to our lives.

## 1 INTRODUCTION

Although the world in the battle against the pandemic has recorded an unprecedented death toll, according to World Health Organization and Our World in Data, still six times more deaths from cardiovascular disease compared with the coronavirus disease 2019 (COVID‐19) are reported each year. Abnormal vital signs, including agonal breathing, indicate a relatively short duration from cardiopulmonary arrest.^1^, ^2^, ^3^ Since the timely detection is followed by time‐dependent actions, which comprise the chain of survival,^4^ the continuous monitoring of vital signs for 24 h is essential to predict and treat in‐hospital and out‐of‐hospital cardiac arrests.^5^

As a fundamental part of the pandemic response, telehealth provides excellent flexibility in healthcare delivery at the most critical time. Constantly measured vital signs, providing the body's most basic functions to medical staff, play a crucial role in implementing telehealth technology, which includes detection of sudden cardiac arrest. Remote medical care benefits individuals, including chronic patients, the elderly, people living in rural and remote areas, immunocompromised, and pregnant.^6^, ^7^ Besides, the measurement of continuous vital signs in daily life leads to accurate diagnosis of chronic disease.^8^, ^9^, ^10^, ^11^, ^12^, ^13^, ^14^, ^15^ Therefore, a wearable device that can safely collect continuous health data for anyone, anytime, anywhere is attracting attention as the most pertinent data‐collecting device for telehealth.^16^, ^17^, ^18^, ^19^, ^20^, ^21^ A keynote speaker in the 2022 Consumer Electronics Show also highlighted the essential role of wearable devices in implementing telehealth.

Several technologies have been proposed that measure some of the vital signs with one sensor and have the potential to be applied to wearable devices: Impedance‐plethysmography (IPG), photo‐plethysmography (PPG), and ultrasound (US) wall tracking. The IPG method measures the action of the heart and lungs with a change in the electromagnetic (EM) field.^22^, ^23^, ^24^, ^25^, ^26^, ^27^ In the IPG method in which the sensor's metal must contact the skin directly, the possibility of electric shock cannot be excluded, and users may also feel unpleasant. The fact that two to four sensors are required also makes the IPG method challenging to be applied to wearable devices. A PPG sensor measures the pulsating volume of blood at the peripheral artery to estimate the cardiac action using light.^28^, ^29^, ^30^, ^31^, ^32^, ^33^, ^34^, ^35^, ^36^ In particular, the dorsal side of the wrist, which is mainly used as a measurement site for a commercial smartwatch, is challenging to expect accuracy in monitoring the normal breathing.^37^ The US method measures the thickness of the blood vessels^38^, ^39^, ^40^, ^41^ to estimate the cardiac actions. We believe no US sensor has been reported to measure pulmonary signals in a wearable form. In the state‐of‐art study, the severe degradation of the ultrasound signal amplitude without gel was one of the remaining problems to be addressed.^42^ Moreover, the vast power consumption of the above methods is a challenge for their effective use in desirable applications.^43^, ^44^, ^45^

High power consumption can lead to overheating of the device, which can cause problems when the device is in contact with the skin for a long time.^46^ Above all, the limited capacity of the battery in wearable devices can restraint the function of the sensors shrinking the monitoring period and run‐time.^47^ The PPG and US sensors consume the most of power as the loss from energy conversion. When energy is converted to a different form, some input energy is turned into disordered energy, such as heat. The PPG and US methods convert the electric energy into light and sonic energy, respectively. When it comes to the energy transformation, the IPG method without the loss has an advantage compared with the others. Frequency also affects the operating power of the devices. The power consumption of the IPG method due to the low operating frequency (100 kHz) can be reduced by raising the frequency to the GHz band and lowering the skin resistance by 80%. In addition, the sensor structure should be optimized for the sensor's materials and input characteristics. The input signal from the fine‐tuned sensor can reduce wasted power by minimizing the signal reflected from nontarget tissues such as skin.

In this article, we propose an ultralow powered multifunctional sensor, which measures all cardiopulmonary vital signs by detecting an electromagnetically reactive near field. The ultralow power consumption of the proposed sensor was implemented with no energy transformation, operation at high frequency (5.4 GHz), and judicious sensor design. Changes in the vascular area due to heartbeat and respiration appear as changes in bioimpedance with different frequency ranges on the wrist.^37^ By monitoring the bioimpedance via the intensity change of the near field continuously, the blood pressure, heart rate, and respiration signal can be measured noninvasively. This small and lightweight sensor is placed on the bare skin of the wrist without the additional intermediate. Since the sensor operates with ultrahigh efficiency at high frequency, it is suitable for continuous monitoring of vital signs for 24 h eliminating the risk of heat generation or electric shock. The proposed ultralow power sensor measuring noninvasive, continuous, and accurate cardiopulmonary vital signs at once will be one of the most promising sensors for a wearable device to bring telehealth to our lives.

## 2 RESULTS

### 2.1 Working principle and device design

**FIGURE 1:**
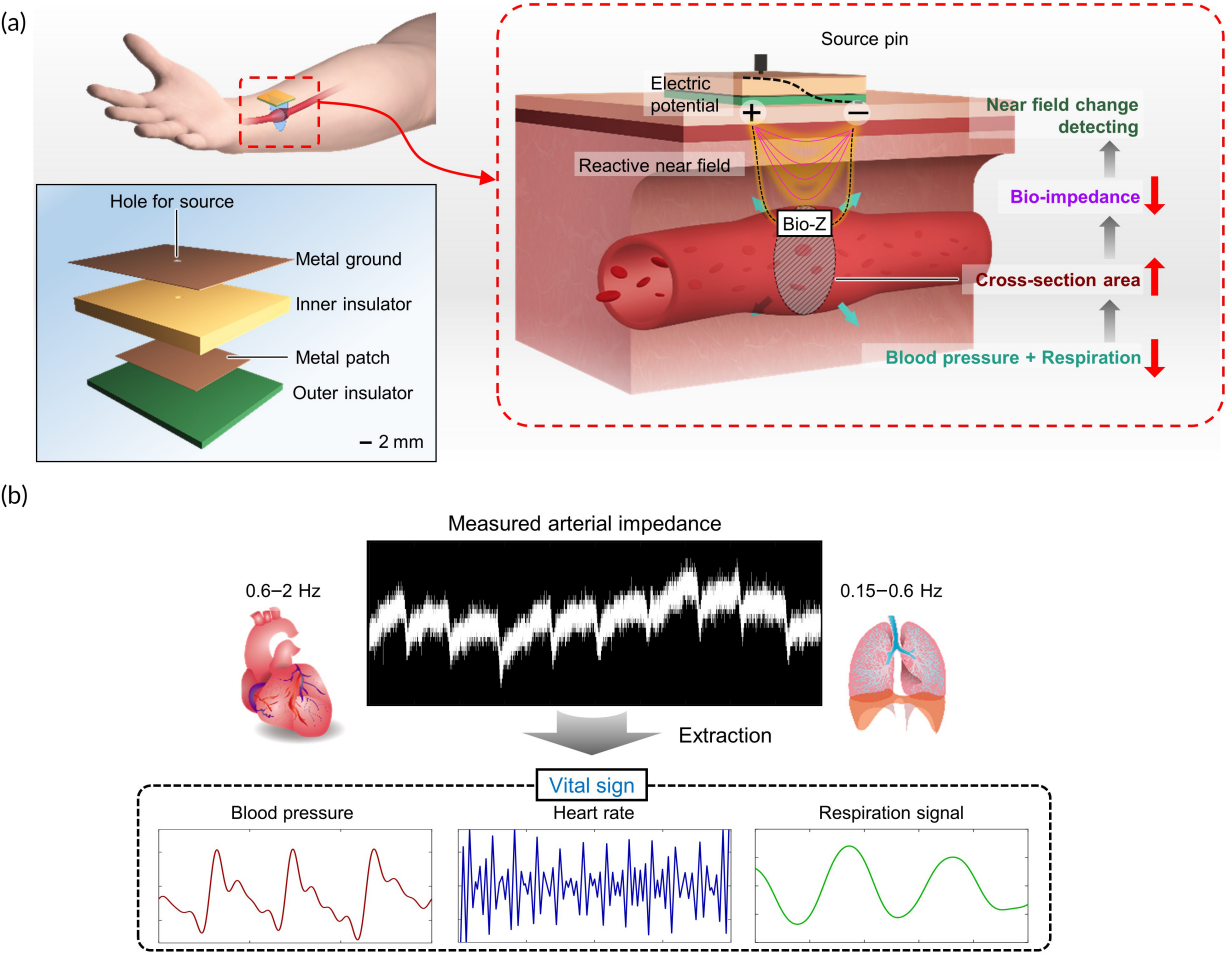
Working principle and the design of an ultralow power vital signal sensor using a reactive near field. (a) Schematics of the vital sensor, with the structures labeled. When an electric potential is applied to the metal layers through the source pin, a reactive near field is emitted near the outer insulator. When this sensor is attached to the wrist, it is possible to detect changes in the cross‐sectional area of the radial artery due to cardiopulmonary action through the reflected field intensity. (b) Bioimpedance converted from sensor measurement can extract target vital signs by different analysis methods in each frequency range.

Cardiac action causes blood to flow through the blood vessel throughout the body. The ejected blood has a pressure that pushes the wall of the vessel and changes the cross‐sectional area (CA) of the artery. At the same time, the respiration signal is modulated to the hemodynamics under the influence of pulmonary arteries. Since the changes in the vascular CA can be expressed in terms of the bioimpedance, multiple cardiopulmonary vital signs can be monitored by measuring bioimpedance (Supporting Note 1). The proposed sensor measures bioimpedance (Bio‐Z) using an EM reactive near field (Figure 1a). The reflected field intensity, which can be converted into bioimpedance through a well‐known equation,^48^ changes in response to the CA variation of the vessel.

The sensor is positioned by targeting the radial artery near the skin of the wrist to improve accuracy and usability. By considering the anthropometry and anatomy of the wrist for the application to the wearable device, the rough dimensions of the sensor were decided (Supporting Note 2). The sensor comprises four layers with the aim of low power consumption and safety. The sensor's structure, imitating an antenna, radiates an EM field power‐efficiently. The ground and patch layers are metal, and the insulators are FR‐4 generally used in antennas or circuits. As the outer insulator is applied between the metal sheet and the skin, the possibility of electric shock is totally excluded. The input power applied at the patch layer through the source pin induces the anode and cathode at both edges of the layer. The electrical potential difference between the opposite nodes generates a reactive near field in the body direction.

The bioimpedance calculated by the reactive near field can be converted into the blood pressure, heart rate, and respiration signal using different analysis methods in each frequency range, as shown in Figure 1b. The pulsatile component (0.6–2.0 Hz) mainly reflects the periodic blood volume changes within each cardiac cycle, and the quasiconstant component is associated with the respiratory (0.15–0.60 Hz).^37^ To observe the detail peaks constituting the pulse wave, we extended the frequency range of the pulsatile component to 5 Hz. Using the demodulation in frequency domain, the proposed sensor can measure three out of the four primary vital signs.

### 2.2 Ultralow power vital sign sensor

The operating frequency and dimensions of the proposed sensor are engaged to form a reactive near field with the minimum loss. An elaborate sensor design to operate at the optimal frequency increases the signal‐to‐noise ratio (SNR), enabling vital sign measurements at a low power level. The human body, including blood vessels, is modeled electromagnetically with the real and imaginary parts of impedance, resistance, and reactance.^49^ The proposed method measures blood pressure by modeling the target body part as pure resistance without reactance.^22^ Since the bioimpedance varies along frequency, various 3D EM simulations (using Ansys HFSS v15.1) were conducted to determine the operating frequency determined as 5.4 GHz (Supporting Note 3).

**FIGURE 2:**
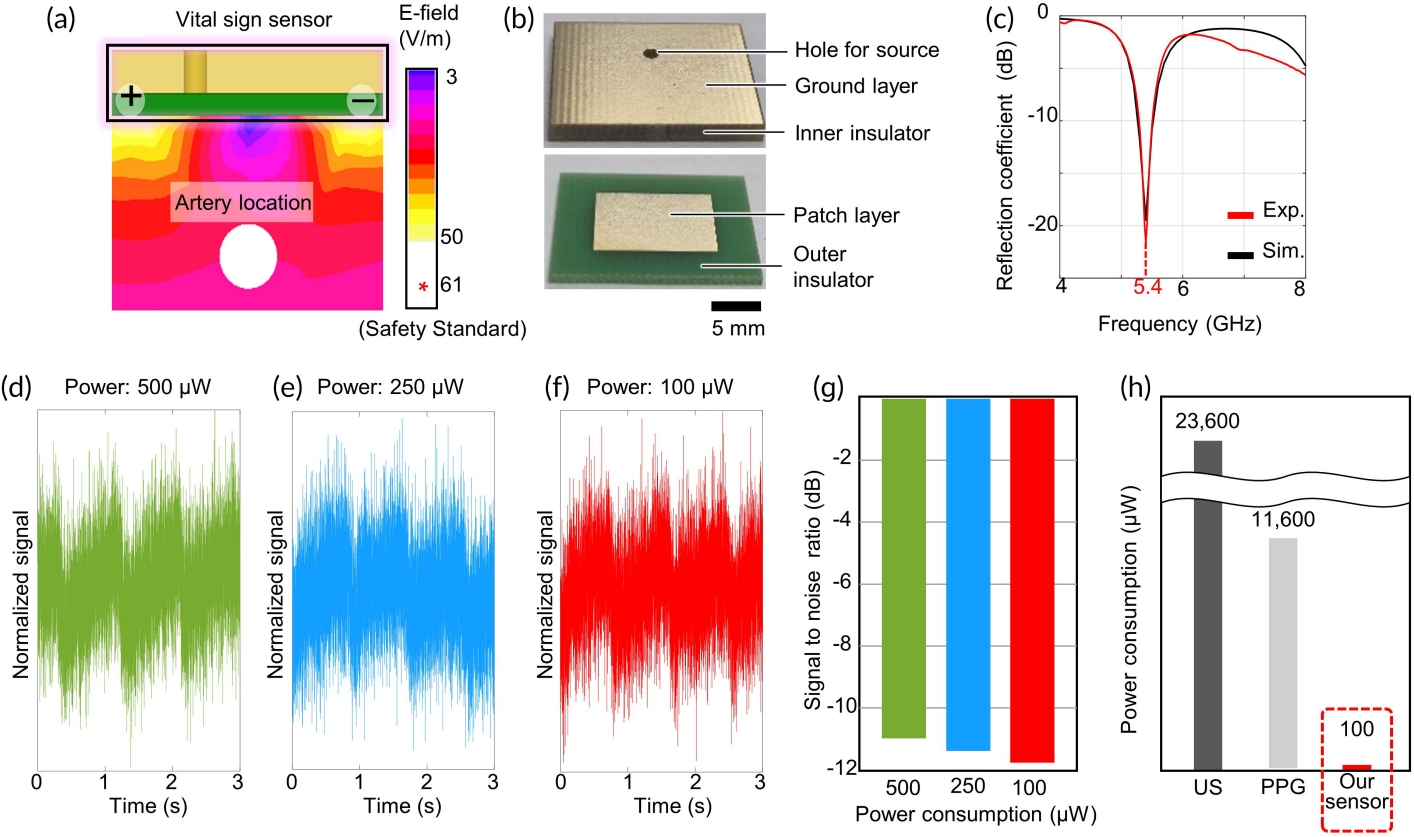
Design, fabrication, and verification of the proposed vital sign sensor performance. (a) The electric field simulation result of the proposed sensor. The maximum intensity of the electric field does not exceed the safety standards.^62^, ^63^, ^64^ (b) The pictures of the fabricated sensor. In the above photo, there is a ground layer and a 2 mm insulator, and in the below one, a patch layer is printed on the green 1 mm insulator. (c) The reflection coefficient graphs from the simulation (black line) and experiment (red line) to check operating frequency. (d–f) The sensed signal results using the powers: 500, 250, and 100 μW, respectively. (g) Signal‐to‐noise graphs of the sensor for various power consumption (500, 250, and 100 μW). (h) Power consumption comparison between the proposed ultralow power vital sign sensor and conventional ones such as ultrasound (US) wall‐tracking,^38^ photo‐plethysmography (PPG; Supporting Note 6), and impedance‐plethysmography.^45^

The size of the patch layer was designed (10.9 × 14.9 mm), and the detail dimensions were also modified delicately according to the operating frequency. Fine‐tuned hole location and overall sensor size allow very high SNR for the sensor. Figure 2a,b depict the results of the simulated and fabricated sensor with the exact lengths. In Figure 2a, the induced opposite poles at both ends of the patch generate an intensive EM field, but the field does not exceed the safety standard (61 V/m for 5.4 GHz; Supporting Note 4). The reflection coefficient, which means the loss of generated EM field along the frequency, is observed in Figure 2c. In the figure, the graphs from the simulation (black line) and measurement (red line) have the perfectly matched resonant frequencies of 5.4 GHz as intended, thanks to the judicious sensor design. Experiments evaluated the SNR for the reliable operation of the sensor at ultralow power. The sensor on the wrist measured bioimpedance with various input powers: 500, 250, and 100 μW for Figure 2d–f, respectively. We can observe that the SNR from the 100 μW input power is comparable to that from the 500 μW (Figure 2g). This suggests that the proposed sensor is fully functional even at ultra‐low power of 100 μW (Supporting Note 5). Despite the low SNRs caused by high operating and sampling frequency, the vital signs can be extracted clearly as the frequency range of the noise is much higher than that of the desired one.

### 2.3 The validation of the proposed sensor: blood pressure waveform

**FIGURE 3:**
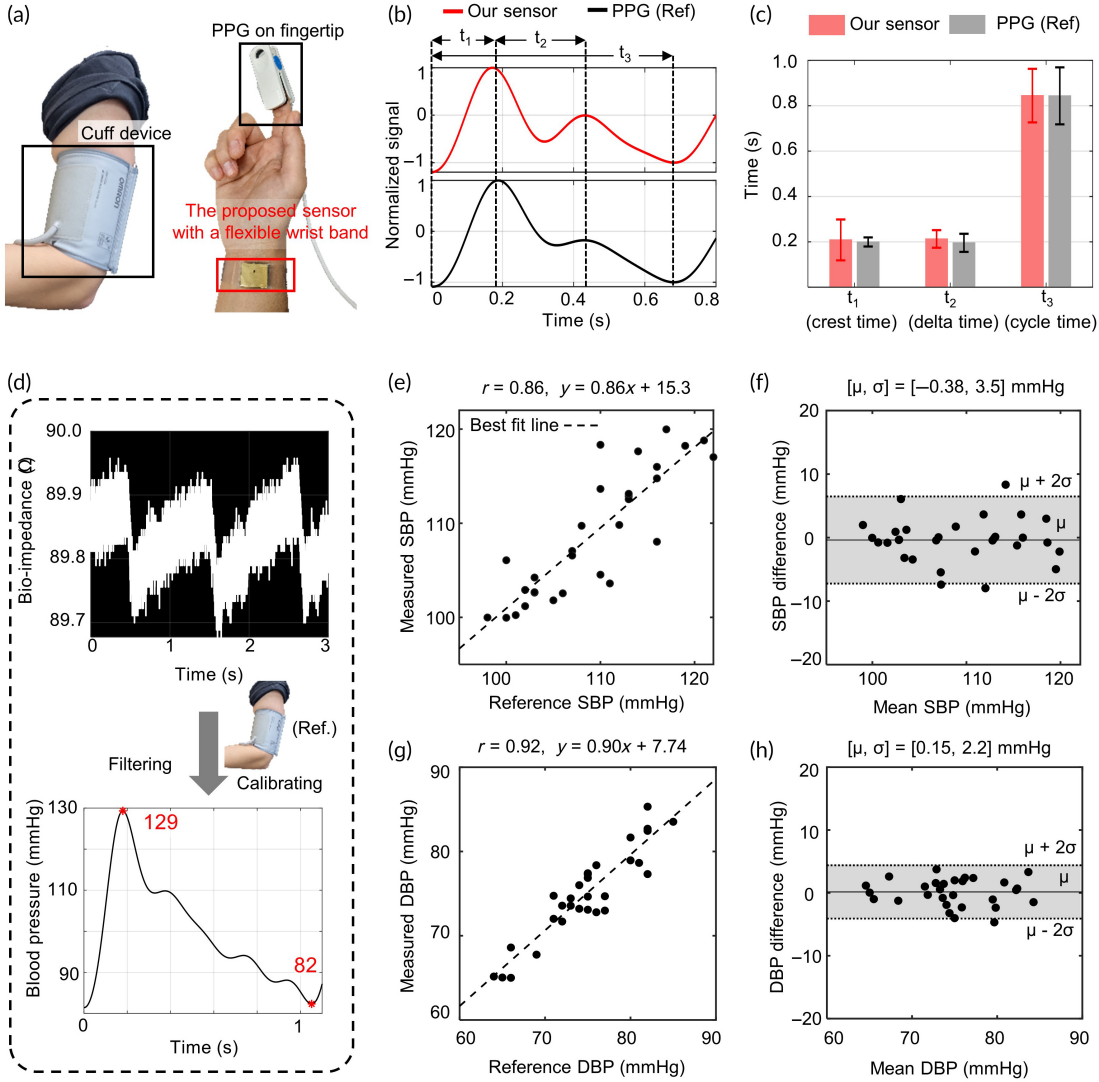
Blood pressure sensing performance results with a photo‐plethysmography (PPG) sensor and a cuff device (*n* = 30). (a) The pictures of the hand‐wearing cuff device, PPG sensor on the fingertip, and the proposed sensor on the wrist. The proposed sensor can be combined with flexible accessories like a wristband. (b) The comparison of the blood pressure waveform from our proposed sensor (red line) on the wrist and the photo‐plethysmograph (black line) on the fingertip. The time gaps, *t*_1_, *t*_2_, and *t*_3_ represent crest time, delta time, and cycle time, respectively. (c) The comparison of the time gaps from our sensor and PPG (*n* = 30). The error bar means ± SD. Only *t*_2_, related to the echo from the periphery (fingertip), shows a statistically significant difference (*p* < 0.05). (d) The observed bioimpedance of the radial artery from the proposed sensor for 3 s. The blood pressure waveform plot is filtered from the measured bioimpedance and calibrated with a cuff device. (e–h) Correlation (e,g) and Bland–Altman plots (f,h) comparing the systolic blood pressure (SBP) and diastolic blood pressure (DBP) from the sphygmomanometer and the proposed sensor. *r*, correlation coefficient; *μ*, bias error (mean of errors); *σ*, precision error (SD of the errors); solid lines in Bland–Altman plots, bias errors; dashed lines in Bland–Altman plots, 95% limits of agreement. US, ultrasound.

The accuracy of the blood pressure from the proposed sensor was validated by comparing the measurement with those from a commercial PPG sensor and an upper arm sphygmomanometer. The experiments were conducted on 30 subjects (age: 27 ± 2 years; height: 169 ± 9 cm; weight: 71 ± 15 kg; 20 males and 10 females). As shown in Figure 3a, the PPG and proposed sensors were worn on the right fingertip and wrist, respectively, and a cuff‐based sphygmomanometer was on the left upper arm.

For the validity check, the waveform features (*t*_1_, *t*_2_, and *t*_*3*_) were compared after normalizing the simultaneous measurements obtained from the PPG and proposed sensors (Figure 3b,c). The *t*_1_, *t*_2_, and *t*_3_ are time gaps between the foot and the first peak (systolic peak), the first and second peaks, and the first and second feet of the waveform, respectively. As shown in Figure 3c, *t*_1_ and *t*_3_ obtained from the proposed and PPG sensors show almost the same values. Conversely, *t*_2_, which is related to the time taken for the pressure wave to propagate from the heart to the periphery and back,^50^ showed a statistically significant difference (*p* < 0.05) and was slightly longer in measurements from the proposed sensor attached to the wrist than those from the PPG sensor attached to the fingertip. Consequently, it was verified that the proposed sensor reflects cardiac actions well compared with a commercial PPG sensor.

On the same subject group, systolic blood pressure (SBP) and diastolic blood pressure (DBP) were calculated based on the bioimpedance measured by the proposed sensor for 10 s using the process depicted in Figure 3d. The reference blood pressures were measured using the sphygmomanometer before and after the experiment and averaged. The correlation and Bland–Altman plots for the SBP and DBP values in rest are illustrated in Figure 3e–h. For both SBP and DBP, the correlation coefficient (*r*) is 0.86 or more. The proposed sensor has bias errors (*μ*) of −0.38 and 0.15 mmHg in SBP and DBP, and the bias errors are statistically insignificant (*p* > 0.05). The precision errors (*σ*) of SBP and DBP are 3.5 and 2.2 mmHg, respectively. The optimal frequency is distributed within 5.4 ± 0.7 GHz depending on the subject (Supporting Note 7). However, it should be noted that all results in this article were measured at 5.4 GHz and the reliability of the proposed sensor was validated, showing comparable bias and precision errors to those of the other cuff devices.^51^, ^52^

### 2.4 The validation of the proposed sensor: cardiac signals before and after exercise

**FIGURE 4:**
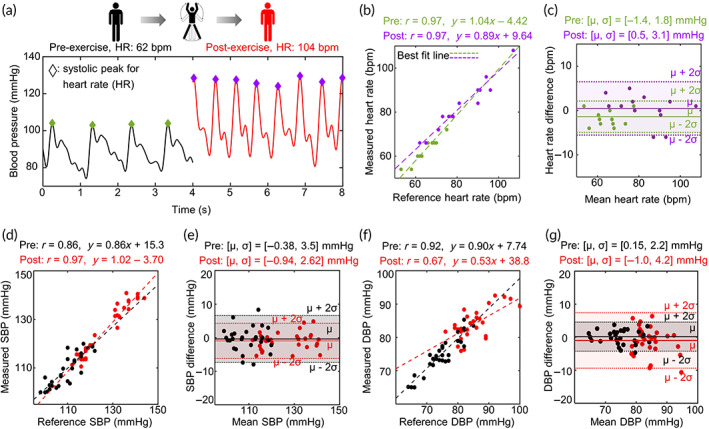
Blood pressure and heart rate measurements comparison before and after exercise (*n* = 30). (a) blood pressure waveform comparison before (black line) and after (red line) exercise. Diamonds mean systolic peaks for heart rate measurements. (b–g) Correlation and Bland–Altman plots comparing the heart rate (b,c), systolic blood pressure (SBP; d,e), and diastolic blood pressure (DBP; f,g) from the sphygmomanometer and the proposed sensor before (black lines and dots) and after (red ones) exercise on the same 30 subjects. *r*, correlation coefficient; *μ*, bias error (mean of errors); *σ*, precision error (standard deviation of the errors); solid lines in Bland–Altman plots, bias errors; dashed lines in Bland–Altman plots, 95% limits of agreement.

The same consented subjects were asked to perform aerobic exercise for 1 min after the first experiment (Figure 4a). The blood pressure was measured immediately after the workout using the same protocol and sensor as the former experiment. The heart rate was calculated by the number of systolic peaks in the blood pressure waveforms, and a comparison with the measurement from the same sphygmomanometer was carried out for performance validation. In Figure 4b–g, the correlation and Bland–Altman plots for heart rate, SBP, and DBP before and after exercise are shown. Data obtained before exercise are marked in green for heart rate and black for blood pressure, and those after exercise are drawn in purple for heart rate and red for blood pressure, respectively. The increases in heart rate and SBP after a workout agree with common sense. It was confirmed that all bias errors at post‐exercise, which are −0.94 mmHg for SBP and −1 mmHg for DBP, were statistically insignificant (*p* > 0.05). In the measurement results during exercise, noisy signals caused by motion artifacts were observed. But, the precision error stays low after exercise in all cases. The analytic results are shown in Table 1, indicating overall blood pressure measurement accuracy of the given monitoring system is categorized as “Grade A (mean absolute difference [MAD] ≤ 4 mmHg)” according to grading criteria set by the Association for the Advancement of Medical Instrument (AAMI) and the British Society of Hypertension (BHS).^53^ It was shown that, even when blood pressure varies and some factors make the measurement environment unstable, the proposed sensor produces comparable results with those of the commercially available cuff device.

**TABLE 1:** Validation of results at preexercise and postexercise.

Parameters of interest	Preexercise		Postexercise	
	SBP	DBP	SBP	DBP
*r*	0.86	0.92	0.97	0.67
*μ* (mmHg)	−0.38	0.15	−0.94	−1.00
*σ* (mmHg)	3.44	2.13	2.57	4.12
MAD (mmHg)	2.45	1.51	2.00	3.04

Abbreviations: DBP, diastolic blood pressure; MAD, diastolic blood pressure; SBP, systolic blood pressure.

### 2.5 The validation of the proposed sensor: respiration signal

**FIGURE 5:**
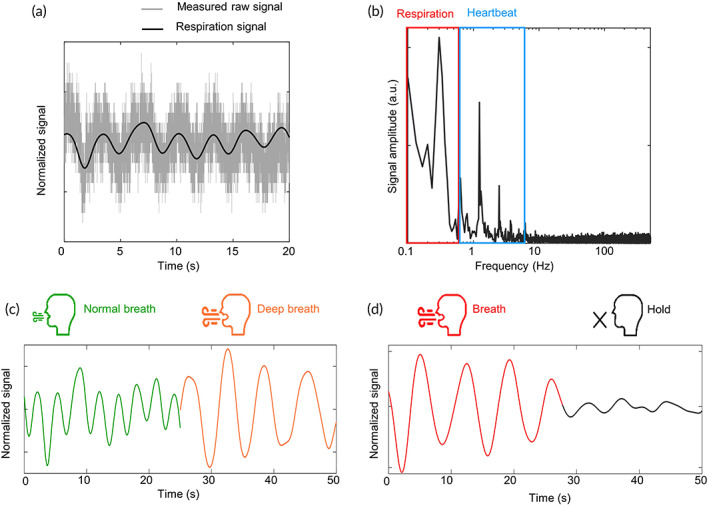
Respiration signal extraction results. (a) The raw cardiopulmonary data from the sensor and the demodulated respiration signal. (b) The measured data in the frequency domain. (c) Respiration signal at normal (green line) and deep (orange line) breaths. (d) Breath‐holding test. Breath is held from 27 s (from red to black line).

Figure 5a illustrates the raw cardiopulmonary data from the sensor and the demodulated respiration signal. The measured data can be analyzed in the frequency domain using Fourier transform, as shown in Figure 5b, and the data in a frequency range of 0.1–0.6 Hz elicits the respiration signal (Supporting Note 8). Three types of breath (normal, deep, and hold) were recorded since respiration could be artificially controlled, unlike cardiac action. The experiment was conducted on a voluntary subject. With expiration, the blood flow to the left side of the heart increases, the left stroke volume increases, and the arterial blood pressure increases. With positive pressure ventilation, the opposite circulatory effects occur.^54^ Thus, we can observe the volume and velocity of the inhaled and exhaled air from the measurements. The normal and deep breathes for 25 s are compared in Figure 5c. For the deep breath (orange line), the extracted signal shows a larger amplitude and long period. The respiratory rates are 19 cycle/min and 10 cycle/min for a normal and deep breath, respectively. The breath‐holding experiment was carried out, and the result is depicted in Figure 5d. The subject started to hold her breath at 30 s approximately (black line), and the signal showed a clear difference with the preceding deep breath (red line). We demonstrated the possibility of monitoring respiratory rate and volume with the proposed sensor.

In Figure 5b, there is an overlapped frequency, that is, 0.6 Hz. The signal around 0.6 Hz can be demodulated into both respiration and cardiovascular signal. A breath cycle of 0.6 Hz means that a person breathes 36 times in a minute. Since a normal human breathes 12–15 times a minute at rest, we can easily imagine the situation is an abnormally nervous condition.^55^ On the other hand, a cardiac cycle of 0.6 Hz, that is, 0.6 beats/s, indicates it takes almost 2 s to complete the cycle, which means the body is extraordinarily relaxed. Assuming a healthy heart and a typical rate of 70–75 beats/min, each cardiac cycle takes about 0.8 s.^56^ The point is which condition the body is under. Generally, it is appropriate to regard the quasiconstant signal around 0.6 Hz as respiration. In the case of the cardiovascular signal, since the power density around 0.6 Hz is lower than that of the main peak around 1 Hz, the low‐frequency signal seems a slow and windless envelope in the time domain (Supporting Note 9).

### 2.6 The robustness of the sensor in the various environments

**FIGURE 6:**
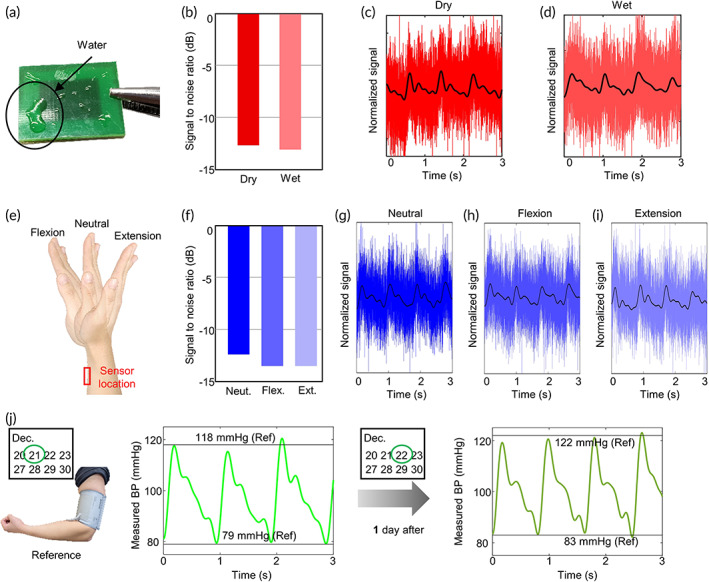
Experiment results for the robustness of the proposed sensor (a) the picture of the sensor with water. (b) Signal‐to‐noise comparison of the sensor with and without moisture. (c,d) Sensed bioimpedance signals at dry (c) and wet (d) conditions. (e) The pictures for the three postures of the wrist. (f) signal‐to‐noise comparison between three different postures of the wrist. (g–i) Sensed bioimpedance signals with postures of the wrist: (g) neutral, (h) flexion, and (i) extension. (j) Experimental blood pressure results for long term without calibration. Blood pressure waveforms at a time and after 1 day. The estimated systolic and diastolic blood pressures are compared with the reference blood pressure from the upper arm‐cuff device. The proposed sensor is calibrated once at the beginning of the experiment.

The proposed vital sign sensor applied to the wearable device should continuously collect the user's health care data even in various circumstances. A volunteer investigated the robustness of the sensor in three usual cases (moisture exposure, measurement site deformation, and long‐term period without calibration). The accuracy of the proposed sensor is confirmed with a dry sensor and neural posture of the wrist in the previous sections. Suppose the SNRs in modified conditions are similar to them in original conditions (dry and neutral). In that case, it can be said that the accuracy of the sensor in various conditions is indirectly verified. SNRs with dry and wet sensors were compared (Figure 6a–d). The sensor was exposed to tap water of 0.2 mL in the wet condition,^38^ which is a sufficient amount compared with the secretion rate at the wrist (<10 nL/min/cm^2^).^57^ The efficiently operating sensor ensures reliable measurements, confirming that the sensor can monitor the vital signs in a moist environment such as sweat. The angle of the wrist can affect the sensor's performance. We observed the quality of the measurements in three postures (neutral, flexion, and extension) of the wrist in Figure 6e–i. The SNRs of the flexed and extended wrists are lower than that of the neutral position in which the sensor tightly contacts the skin, but the results still assure clear vital signs. Like other devices for monitoring vital signs, the proposed sensor requires initial calibration to estimate the absolute blood pressure value. After the initial calibration, the volunteer wore the sensor for 6 h to check whether the accuracy was maintained for a long time. Figure 6j shows the comparison between the measurements from the proposed sensor and the cuff device at the beginning and end of the experiment. There was no additional calibration within 24 h. There was a little increase of the root‐mean‐square error on Day 2 (5.6 mmHg) compared with that on Day 1 (3.4 mmHg), indicating that the proposed sensor maintained high accuracy without additional calibration even after a long time.

## 3 DISCUSSION

Cardiovascular diseases take an estimated 17.9 million lives each year, six times higher than COVID deaths. Moreover, only 0.07% of patients with out‐of‐hospital cardiac arrest are treated with emergency medical services.^58^ These indicate and emphasize the importance of the 24 h monitoring of vital signs. Telehealth using wearable devices with vital sign sensors is one of the solutions by delivering medical services to the patients in remote sites at the most critical time. Here, we introduced an ultralow power vital sign sensor that can be applied to wearable devices to realize telehealth. The fabricated sensor generates a reactive near field to monitor changes in the CA of the radial artery in which the signals of circulation and respiration are modulated. Therefore, the proposed sensor can acquire three cardiopulmonary vital signs (blood pressure, heart rate, and respiration signal) by fundamental principles and straightforward data processing. The proposed method carries out measurements without converting electrical energy into other energy forms, and the sensor structure is fine‐tuned at the optimal frequency. These features lead to the drastic decline of the power consumption of the sensor. Twenty‐four hours constant monitoring based on the proposed ultralow power sensor, unlike the other sensors that support the abnormality's periodic check, assures that cardiopulmonary arrest can be an expectable and responsive event (Supporting Note 6).

The performance of the proposed sensor was evaluated compared with the commercial upper arm cuff device and PPG sensor, and we could observe the reliable vital signs in various circumstances in daily life. The rigid material used in the sensor is very resistant to damage so that it can be used semi‐permanently without replacement. Even after prolonged wear, there was no skin sensitization (Supporting Figure 6). However, the proposed flat sensor lacks accessibility to the skin (Supporting Figure 12), which can lead to frequent calibration. The additional ergonomic design will enhance calibration duration and accuracy of measurement. The high degree of freedom in selecting materials and shapes is another strength of the proposed sensor, as delicate sensor design is possible based on the elemental principles according to the new structure and material (Supporting Note 10).

Since the experiment conducted in this article is at the laboratory level, further research is needed to go to the anyone, anytime, and anywhere wearable sensor. For example, a circuit module design study that replaces the existing measurement equipment with a large volume is expected (Supporting Note 11). The integration of the sensor, measurement circuit, and battery will prove the sensor performance for massive users in various environments, that is, tracking blood pressure during the entire day. We believe that our vital sign sensor is one of the most promising devices to accomplish the prevalence of telehealth using wearable devices and contribute the flexible healthcare delivery, even at the most crucial moment.

## 4 MATERIALS AND METHODS

### 4.1 Material of the sensor

The proposed sensor consists of two metal layers and two insulator layers. The material of the metal layer is copper with a thickness of 22um and a Young's modulus of 120 GPa. The material used for the insulator is FR‐4, which is used as a printed circuit board. FR‐4 is composed of several layers of glass fibers impregnated with epoxy resin and has a Young's modulus of 12.4 GPa.

### 4.2 Study design

We investigated the feasibility of an ultralow power vital sign sensor monitoring the continuous blood pressure, heart rate, and the respiration signal noninvasively by generating the reactive near field. An Institutional Review Board (IRB) approval was received from the Ulsan National Institute of Science and Technology (UNISTIRB‐21‐31‐A). Note that this is not an IRB approval of a hospital because the general public can use the devices in the absence of medical experts. Subjects were recruited by targeting male/female adults with healthy bodies. Informed consent was obtained after explaining the process of the experiment and the possible indications. A sensor development experiment (*n* = 2) was conducted before the subject experiment. The subject experiment (*n* = 30) was conducted using the same protocol. The number of subjects was based on a similar study for developing a blood pressure monitor.^59^ There were no outliers in the experimental results.

### 4.3 Experimental environment and protocol

The subjects participating in the experiments were informed about the topic of this study. After that, those who gave written informed consent were seated in a comfortable position in front of an experiment desk. An upper arm sphygmomanometer (Omron HEM‐7121) was worn on the left, and the proposed sensor was applied to the right wrist using hypoallergenic medical tape. For calibration, measurement using the proposed sensor was performed for 10 s after measuring the blood pressure with the cuff‐type device. The proposed sensor was connected to a network analyzer (KEYSIGHT PNA Network Analyzer N5222A), which can read high‐frequency data with a coaxial cable and is controlled through a desktop. After measuring the blood pressure and heart rate with the cuff‐type device, a commercially available PPG sensor (PhysioLab PSL‐iPPG2‐C) was fixed to the index finger of the right hand. The PPG sensor was connected to the desktop through a microcontroller unit (Arduino Uno [R3]) to collect data. In the first experiment, while measuring with the proposed sensor for 10 s, data was acquired with the PPG sensor. The second experiment was conducted with the same 30 subjects who consented to exercise between the experiments. Aerobic exercise affecting the heart rate was performed for 1 min, and blood pressure and heart rate were measured right after exercise with the same protocol as in the first experiment. A volunteer conducted experiments to collect respiration signals seated in the same comfortable position. The identical sensor used in the previous experiments was worn on the same site. The validations on the sensor robustness under various conditions (moisture, angle, time) were carried out similarly for the voluntary individual who gave consent.

### 4.4 Data processing

#### 4.4.1 Blood pressure and heart rate

All data measured by the PPG and the designed sensors were filtered by a band‐pass filter with a 0.6–5 Hz passband before analysis. This removed low‐frequency signals caused by breathing, high‐frequency signals, and random noise from the surrounding environment and the measuring equipment. The heart rate was calculated based on the number of systolic peaks of the pulse waveform. An ensemble averaging was employed to merge the 10 s of data into one period.

The designed sensor estimates blood pressure using the relationship between blood vessels' CA and bioimpedance. The relationship between the CA of the blood vessels and the blood pressure can be found in Equation (1).^60^

(1) $$P\left(t\right)=P_{D}e^{\alpha \left(\left(\mathrm{CA}\left(t\right)\right)/\left({\mathrm{CA}}_{D}\right)-1\right)}$$

Since the bioimpedance is inversely proportional to the CA at the operating frequency, Equation (1) is converted as follows:

(2) $$P\left(t\right)=P_{D}e^{\alpha \left(\left(Z_{D}\right)/\left(Z\left(t\right)\right)-1\right)}$$

where $Z_{D}$ is the impedance at the DBP, and $Z\left(t\right)$ is the impedance as a function of time.

The constants α, $P_{D}$, and $Z_{D}$ in Equation (2) complete the algorithm by converting the measured bioimpedance data into blood pressure data. Using the DBP and the SBP data measured by the cuff device and the proposed sensor for the calibration, the unknown constants can be determined along the following equation.

(3) $$\alpha =\ln\left(\frac{P_{S}/P_{D}}{Z_{D/}Z_{S}}-1\right)$$

#### 4.4.2 Respiration signal

Measurements from the proposed sensor went through a 0.1–0.6 Hz band‐pass filter.^61^ After that, the window moving average with a Gaussian filter smoothed the signals.

#### 4.4.3 Signal‐to‐noise ratio

SNR refers to comparing the level of the desired signal with that of background noise. SNR is defined as the ratio of signal power to the noise power, expressed in decibels like Equation (4):

(4) $$\mathrm{SNR}=10\log\left[\frac{P_{\text{desired}}}{P_{\text{noise}}}\right]$$

### 4.5 Statistics

The pulse waveform was simultaneously measured using the proposed sensor and the current PPG sensor to verify the validity of the blood pressure monitoring of the proposed sensor. It was confirmed whether the differences in the two data pairs were statistically significant using the paired *t*‐test. Standard analyses were used to assess the heart rate, DBP, and SBP measurements from the proposed vital sign sensor against the reference measurements from the cuff device. The accuracy was shown visually using the correlation and Bland–Altman plots. The correlation coefficient (*r*), bias error (*μ*), and precision error (*σ*) were used to evaluate the accuracy. It was also verified that all bias errors were statistically insignificant with the high *p*‐values (*p* > 0.05). The accuracy was also validated with MAD calculated by Equation (5).

(5) $$\mathrm{MAD}=\frac{\sigma^{2}+\mu^{2}}{\sqrt{2\sigma^{2}+\mu^{2}}}$$

## AUTHOR CONTRIBUTIONS

**Seoktae Seo:** Conceptualization (equal), data curation (equal), formal analysis (equal), investigation (lead), methodology (equal), resources (equal), software (supporting), validation (equal), visualization (equal), writing – original draft (equal), and writing – review and editing (equal). **Hyunkyeong Jo:** Conceptualization (equal), data curation (equal), formal analysis (equal), investigation (supporting), methodology (equal), resources (equal), software (lead), validation (equal), visualization (equal), writing – original draft (equal), and writing – review and editing (equal). **Jungho Kim:** Writing – review and editing (supporting). **Bonyoung Lee:** Writing – review and editing (supporting). **Franklin Bien:** Funding acquisition (lead), project administration (lead), and supervision (lead).

## CONFLICT OF INTEREST STATEMENT

The authors have no conflicts of interest to declare.

### PEER REVIEW

The peer review history for this article is available at https://publons.com/publon/10.1002/btm2.10502.

## Supporting information


**Data S1:** Supporting information.

## Data Availability

The data that support the findings of this study are available from the corresponding author upon reasonable request.
