# Supplementary material for: An ultralow power wearable vital sign sensor using an electromagnetically reactive near field
Source: Bioeng Transl Med. 2023 Feb 26;8(3):e10502. doi: 10.1002/btm2.10502 (PMC10189444; doi:10.1002/btm2.10502)
Supplement: Supplementary file 1 — Data S1: Supporting information. [file BTM2-8-e10502-s001.docx]

Supporting information

An ultra-low power wearable vital sign sensor using an electromagnetically reactive near field

**Contents**

Supporting Note 1. Bio-impedance model of the artery………………………………………………………2

Supporting Note 2. Expected sensor location on the wrist………………………………………………...…2

Supporting Note 3. Operating frequency for the proposed sensor…………………………………………..2

Supporting Note 4. Safety standard for the sensor using a reactive near field…………………………..…2

Supporting Note 5. Optimal power consumption for the sensor…………….…………………………….…3

Supporting Note 6. Comparison of power consumptions of vital sign sensors……………………………..3

Supporting Note 7. The optimal frequencies for each subject………………………………………………3

Supporting Note 8. Demodulation process of respiration signal …………………………………………….3

Supporting Note 9. A peak around 0.6 Hz ……………………………………………………………………..3

Supporting Note 10. Thickness and rigidity of the sensor ………………………………………………….4

Supporting Note 11. The block-diagram of the miniaturized measurement circuit……………………….4

Supporting Note 12. Variant coefficient α …………………………………………………………………….5

Supporting Note 13. The correlations between impedance and BMI………………………………………5

Supporting Note 14. The reproducibility of the sensor……………………………………………………….5

Supporting Note 15. The performance of the proposed sensor fabricated with flexible materials………5

Supporting References………………………………………………………………………………………….6

Supporting Figures………………………………………………………………………………………………7

Supporting Tables………………………………………………………………………………………………15

Supporting Videos……………………………………………………………………………………………...16

**Supporting Note 1. Bio-impedance model of the artery:**

The components that make up the body have different electrical properties, including conductivity and permittivity^1–5^. As the effect of permittivity at high frequency becomes insignificant (reactance, X), a blood vessel can be expressed as a structure with a resistive bio-impedance (R) in which the conductivity (σ) is dominant (**Supporting Figure 1**). The resistive bio-impedance of the vessel is calculated using its constant length, conductivity, and time-varying cross-sectional area (CA). Since the conductivity changes very slowly compared to the CA, it seems constant in the high-speed data processing. Thus, without reactance, the bio-impedance of the artery is only inversely proportional to the CA.

**Supporting Note 2. Expected sensor location on the wrist:**

The sensor is positioned to improve accuracy and usability by targeting the radial artery near the wrist's skin (**Supporting Figure 2 a**). The distal length (parallel to the arm) of the sensor is limited to the width of the strap of a watch, which is typically 15 mm. There is a 20 mm space between the radius and radial tendons, and the radial artery at the wrist is located in this space. The lateral length (perpendicular to the arm) was set to 20 mm to locate the sensor on the radial artery.

Each composition of human body such as muscle, fat, tendon, and ligament have individual electromagnetic properties. Since the proposed device is based on the electromagnetic signal, the various measurement site (forehead, finger, toe, earlobe, etc.) with different compositions can affect to the results. The given device is designed to be located on the wrist where the pulsation of radial artery can be detected with the least disturbance by the surrounding tendons, muscles, and tissues. In the **Supporting Figure. 2**, a forearm is modeled approximately for simulation in 3-D electromagnetic simulator (ANSYS HFSS v15.1). As mentioned in Supporting Note 1, the impedance (Z) is a combination of real resistance (R) and imaginary reactance (X). Since the imaginary part increases the magnitude of the impedance and distort the measured signal, the operating frequency of the sensor is determined as 5.4 GHz, where the reactance is zero. On this reason, the proposed sensor can only work properly on the wrist to measure the accurate vital signs. It is possible to measure within a small range (about 2~3mm) around the fixed position. Although the measured signals from the other locations seem similar to the signal from the wrist by accident, validity and reliability of the data cannot be guaranteed.

**Supporting Note 3. Operating frequency for the proposed sensor:**

The CA of the artery can be accurately measured, as the permittivity part of the bio-impedance (reactance) of the wrist is close to 0 with the sensor position and size. Using three-dimensional (3-D) electromagnetic (EM) simulation (Ansys HFSS v15.1), bio-impedance at a determined sensor location on the modelled wrist (**Supporting Figure 2 b**) was obtained. **Supporting Figure 2 c** plots simulated reactance against the frequency, where the absolute values are significant at low and high frequency due to high capacitive and inductive components, respectively. The sensor's operating frequency is decided to 5.4 GHz, where the reactance is nearly zero.

**Supporting Note 4. Safety standard for the sensor using a reactive near field:**

The proposed high-frequency electromagnetic field device is attached to the user's wrist to non-invasively and continuously monitor blood. Since electromagnetic fields are incident directly into the human body, guidelines for electromagnetic field exposure must be satisfied. We simulated propagated electric and magnetic fields of the system using 3-D EM simulation software (ANSYS HFSS v15.1) and verified whether those field intensities satisfy the Standard approved by the Institute of Electrical and Electronics Engineers (IEEE)^6^ and guidelines provided by the International Commission on Non-ionizing Radiation Protection (ICNIRP)^7, 8^. The safety criteria from ICNIRP include the electric and magnetic field intensity, magnetic flux density, and power density, while the criterion for IEEE only deals with the power density at the operating frequency. The simulation results for safety validation of the device are shown in **Supporting Figure 3**. It is confirmed that all safety standards are satisfied, as shown in **Supporting Tables 1 and 2**.

**Supporting Note 5. Optimal power consumption for the sensor:**

To determine the optimal power consumption of the proposed sensor, the signal quality according to several input powers was evaluated with signal to noise ratio (SNR). A sensor was applied to the wrist's skin above the radial artery, and bio-impedances were measured. As shown in **Supporting Figure 4**, the SNR of 100 μW as the operating power is around -10 dB, but that of 50 μW is not even -15 dB. Therefore, the adequate power consumption of the proposed vital sign sensor is determined as 100 μW.

**Supporting Note 6. Comparison of power consumptions of vital sign sensors:**

The world's best-selling commercial smartwatch (Apple Watch Series 5) has a battery capacity of 296 mAh, and the rated voltage is typically 3.7 V. Then, the energy capacity is calculated as 1100mWh. We can estimate the power consumption of the photo-plethysmograph (PPG) sensor considering the available time of the device with (11 h) and without (18 h) monitoring the heart rate. The operating power of the only smartwatch is 61 mW, and the PPG sensor consumes an additional 39 mW. Since the proposed ultra-low power vital sign sensor consumes only 0.1 mW, it can still be used for 18 h even if the sensor is continuously used.

**Supporting Note 7. The optimal frequencies for each subject:**

For the design of the proposed sensor, many 3-D electromagnetic field analysis simulations were performed. The simulations included a detailed model of the wrist, but the structure and electromagnetic properties of the human wrist were expected to be slightly different from person to person. Different wrist models may change the optimal frequency of the sensor. To investigate this, in the first experiment, the optimal frequency was observed using 21 signals increasing by 0.1 GHz from 4.5 GHz to 6.5 GHz for each subject. For 13 of 30 subjects, the optimal frequency was 5.4 GHz, and the optimal frequency distribution graph is shown in **Supporting Fig. 5.** Although the optimal frequency showed variation, it is noted that all experimental results in this paper were measured at 5.4 GHz, the proposed working frequency.

**Supporting Note 8. Demodulation process of respiration signal:**

The data processing session on the paper gives a brief methodology of obtaining respiration signal from the raw data. The raw data are recorded from the subjects in real-time, i.e., in time domain. The band-pass filter, however, is a frequency-selective method that is used to limit the spectrum of a signal to some specified band of frequencies^9^. Therefore, the raw data which is a function of time should be converted into a function of frequency by the Fourier transform before passing the band-pass filter. The **Supporting Fig. 7(a) and (b)** show the data from a subject in raw and after band-pass filtered.

At rest, a normal human breathes 12–15 times a minute, which means 0.2–0.25 Hz^10^. Since we want to cover from the deep breath to the fast abnormal breath, the frequency range is extended (0.1–0.6 Hz). The filtered data in frequency domain is reconstructed into a time function by the inverse Fourier transform. Then the signal contains only the quasi-constant component which is associated with the respiration. A smoothing technique such as window moving average with Gaussian filter is applied in final stage of the processing (**Supporting Fig. 7(c)**).

**Supporting Note 9. A peak around 0.6 Hz:**

A breath cycle of 0.6 Hz means that a person breathes 36 times in a minute. Since a normal human breathes 12–15 times a minute at rest, we can easily imagine the situation is abnormally nervous condition^10^. On the other hand, a cardiac cycle of 0.6 Hz, i.e., 0.6 beat per sec, indicates it takes almost 2 sec to complete the cycle, which means the body is extraordinarily relaxed. Assuming a healthy heart and a typical rate of 70 to 75 beats per minute, each cardiac cycle takes about 0.8 second^11^.

The signal around 0.6 Hz can be demodulated into both respiration and cardiovascular signal. The point is which condition the body is under. Generally, it is appropriate to regard the quasi-constant signal around 0.6 Hz as respiration. In case of cardiovascular signal, since the power density around 0.6 Hz is lower than that of the main peak around 1 Hz, the low frequency signal seems a slow and windless envelope in time domain.

**Supporting Fig. 8** shows the identical graph in Fig. 5b without boxes. **Supporting Fig. 9** is the reconstructed signal in time domain after passing through the band-pass filter from 0.1 to 5 Hz. The modulated signal in **Supporting Fig. 9** can be demodulated by band-pass filter with different cut-off frequency ranges. We tried to compare the difference between those with and without data around 0.6 Hz in **Supporting Fig. 10 and 11** which are demodulated respiratory and cardiovascular signal, respectively. There seems to be small differences but not significant to interpret the data.

**Supporting Note 10. Thickness and rigidity of the sensor:**

A certain thickness of the sensor is necessary to generate designed electromagnetic field with predetermined materials. Young’s modulus, the physical property related to rigidity, is high when the material is hard. On the contrary, flexible material has a lower value of Young’s modulus^13^. An FR-4 used for the insulator layer of the proposed wearable sensor has 12.4GPa^14^ of Young’s modulus which means hard material. The thickness and rigidity of the sensor was barely considered due to its very small size and lightweight, and, among 30 subjects, no subject complained of discomfort during the 30-minute experiment. In a long-term (6 hours) experiment, the sensor did not irritate the skin surface (**Supporting Figure 6**). A small air gap between the attached sensor and the skin (**Supporting Fig. 12**) hardly affects the accuracy of the sensor, since an electromagnetic field used as monitoring blood pressure propagates freely in the air, which gives our sensor a strength compared to other devices using ultrasonic and direct current.

The proposed working principle can apply various materials to the sensor, so flexible material adaptable for body curvature is possible for the sensor. **Supporting Fig. 13** shows the fabricated sensor with a flexible material (Eco-flex 0050, Smooth-On) replacing the original FR-4 material. Young’s modulus of the flexible one is 263kPa^15^ which is a 1/50,000 value of the FR-4. In **Supporting Fig. 13b – c**, the flexibility of the new one is verified, and the sensor can be attached to the skin more intimately without an air gap. The copper sheet, the medium of the ground and the patch layers has 120GPa of Young’s modulus. However, the thickness of the sheet is only 22µm, so copper sheets aren’t too difficult for the sensor to be attached to a slightly curved wrist.

**Supporting Note 11. The block-diagram of the miniaturized measurement circuit:**

The measurement equipment used in the experiment in this paper is too bulky, so it needs to be miniaturized to the circuit size level to become an anyone, anytime, and anywhere (AAA) wearable device. Here, we present a block diagram of a miniaturized measurement circuit for a blood pressure monitoring sensor under study (**Supporting Fig. 14**). The proposed sensor calculates the impedance by measuring S_11_, the ratio of incident signal power to reflected signal power. The circuit should measure and divide the two signal power to get S_11_. We list the functions of blocks in order of S_11_ measurement method.

1. The micro control unit (MCU) commands the phase locked loop (PLL) to generate a signal of the target operating frequency and power.
2. The PLL generates the signal desired by the MCU and sends it to the Radio frequency amplifier (RF amp).
3. The RF amp amplifies the signal and sends it to the circulator.
4. The circulator is an electronic passive device with three ports (ports 1, 2, and 3). Port 1 only sends signals to port 2, and port 2 only sends signals to port 3. The signal from the RF amp goes to port 1 of the circulator and is transmitted to the antenna through port 2.
5. The signal arriving at the antenna goes to the measuring part and is reflected.
6. The reflected signal goes to port 2 of the circulator and goes to port 3.
7. The RF detector outputs the intensity of the reflected signal power in the form of an analog voltage.
8. Analog to digital converter (ADC) converts an analog voltage signal into a digital signal containing information about the magnitude of the reflected signal.
9. The MCU finally derives S_11_ by calculating the commanded incident power level and the digital signal converted by the ADC.

**Supporting Note 12. Variant coefficient α:**

The coefficient $\alpha$, a compensation parameter for blood pressure measurement in the equation (1) and (2), is related to the distensibility of the blood vessel. The vessel distensibility is one of the quantifications of the mechanical properties of the arterial wall^12^. The mechanical properties of the vessel are affected by structural properties of itself or surrounding tissues. Thus, the coefficient $\alpha$ can vary from time to time, from person to person, and from location to location of measurement. That’s why the calibration is so important to the wearable devices that measure the blood pressure in a similar manner such as impedance-plethysmography, photo-plethysmography, and ultrasound wall tracking including our method. On this reason, the duration of the calibrated parameters on the proposed device is experimented and Fig. 6j in the paper shows the result of the validation. The measured blood pressures agree with the reference pressures from the commercial device after 24 hours since fitting parameters determined once at the beginning of the experiment. Since we read the echo from the arterial wall using the electromagnetic field to measure the blood pressure and respiratory signal, it is inevitable that the coefficient $\alpha$ affects to the observed result. In this paper, however, we did not offer absolute values for the respiration signal but the patterns of breath, which means the respiratory results in the paper are hardly affected to the coefficient $\alpha$.

**Supporting Note 13. The correlations between impedance and BMI**

The correlations between the measured impedances and body mass index (BMI) were verified with Pearson correlation coefficient (r). The coefficient between systolic and diastolic impedances and BMI were 0.22 and 0.23, respectively.

**Supporting Note 14. The reproducibility of the sensor**

Blood pressure was measured repeatedly at short intervals to check the reproducibility of the sensor. Since the proposed sensor does not apply pressure to the human body, successive and repeatable observation is possible, unlike the usual sphygmomanometer. We conducted measurements at different times of the day, and there were five trials per measurement. An identical sensor was used for both measurements, and the sensor was calibrated once before each measurement. People can see that the successive trials drive similar and stable results in **Supporting Fig. 15.** The largest standard deviation between five trials at different times was 1.3 mmHg and the smallest one was 0.2 mmHg.

**Supporting Note 15. The performance of the proposed sensor fabricated with flexible materials**

The flexible materials were implemented in the proposed sensor to improve the accessibility to the skin. Since the sensor is applied to the wrist, the wrist is modeled as a circle based on wrist circumstances of the 10^th^ and 90^th^ percentile of females and males, respectively (**Supporting Fig. 16** and Supporting Table 3)^16^. Two plates that have lengths of chords of curved sensors were made to make the measurement unchallenging. As shown in **Supporting Fig.17**, the wrist of the male in the 90^th^ percentile has a low curvature than that of the female in the 10^th^ percentile.

The resonant frequency of the fabricated flexible sensor places near 5.4 GHz, indicating that further tuning of the sensor dimension is required in **Supporting Fig. 18** The curved sensor appears as if the electromagnetic length is shorter than the physical length. That’s why the higher curvature the sensor has, the higher the resonant frequency gets. Although the frequencies shift, the performance of the sensor is not significantly affected because the resonance frequency is still located near 5.4 GHz. The performance of the sensor is roughly checked for one male subject with three repeated measurements (**Supporting Fig. 19**). Bias errors and precision errors are 1.52 and 0.59 mmHg for systolic blood pressure and 4.58 ad 0.2 mmHg for diastolic blood pressure.

**Supporting References**

1. Cole, K. S., Cole, R. H. Dispersion and absorption in dielectrics I. Alternating current characteristics., J. Chem. Phys. 9, 341–351 (1941).

2. Gabriel, C. “Compilation of the dielectric properties of body tissues at RF and microwave frequencies” (Tech. Rep. N.AL/OE-TR-1996-0037, Brooks Air Force Base, 1996).

3. Gabriel, C., Gabriel, S., Corthout, E. The dielectric properties of biological tissues: I. Literature survey, Phys. Med. Biol. 41, 2231–2249 (1996).

4. Gabriel, S., Lau, R. W., Gabriel, C. The dielectric properties of biological tissues: II. Measurements in the frequency range 10 Hz to 20 GHz, Phys. Med. Biol. 41, 2251–2269 (1996).

5. Gabriel, S., Lau, R. W., Gabriel, C. The dielectric properties of biological tissues: III. Parametric models for the dielectric spectrum of tissues, Phys. Med. Biol. 41, 2271–2293 (1996).

6. IEEE-SA Standards Board, “IEEE draft standard for safety levels with respect to human exposure to electric, magnetic and electromagnetic fields, 0 Hz to 300 GHz” in IEEE PC95.1TM/D3.5 (IEEE-SA Standards Board, 2018), pp. 1–6.

7. Herbertz, J. Comment on the ICNIRP guidelines for limiting exposure to time-varying electric, magnetic, and electromagnetic fields (up to 300 GHz). Health Phys. 75, 493–511 (1998).

8. International Commission on Non-Ionizing Radiation Protection, Guidelines for limiting exposure to time-varying electric and magnetic fields (1 Hz to 100 kHz). Health Phys. 99, 818–836 (2010).

9. Haykin S Moher M., “Communication Systems”. 5th ed. Hoboken N.J: John Wiley & Sons; 2009. P. 8–70

10. Barrett KE, Barman SM, Boitano S., “Ganong’s Review of Medical Physiology”, 25e. McGraw Hill; 2018. P. 619

11. Gersh, Bernard J (2000). Mayo Clinic Heart Book. New York: William Morrow. pp. 6–8

12. Arndt JO, Klauske J, Mersch F. The diameter of the intact carotid artery in man and its change with pulse pressure. Pflugers Arch, 1968;301:230–240.

13. Jastrzebski. Nature and properties of engineering materials. NY: Wiley; 1959.

14. Ridout S, Dusek M, Bailey C, et al. Assessing the performance of crack detection tests for solder joints. Microelectron Reliab. 2006;46(12):2122-2130.

15. Ahmed A. Design, Modeling, Fabrication and Testing of a Piezoresistive-Based Tactile Sensor for Minimally Invasive Surgery Applications. M. S. thesis, Concordia University, 2012

16. Gordon, C.C., et al. (2014) 2012 Anthropometric Survey of U.S. Army Personnel: Methods and Summary Statistics. Technical Report Natick/TR-15/007, U.S. Army Natick Soldier Research and Engineering Center, Natick.

**
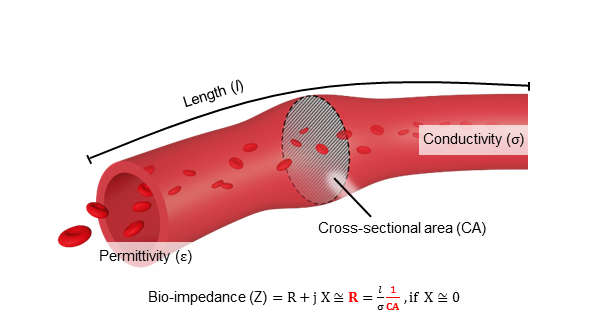
Supporting Figures**

**Supporting Figure 1. Bio-impedance model of the artery with the conductivity and permittivity**

**
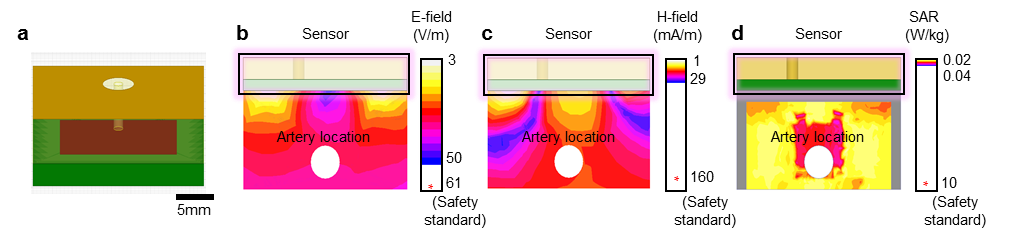
**

**Supporting Figure 2. Optimal operating frequency determination. a,** the wrist model with the expected strap location and sensor location. **b,** the wrist model for 3-D electromagnetic simulation. The port is assigned on the expected sensor location **c,** simulated reactance results. There is no reactance at 5.4 GHz which is determined for operating frequency.

**
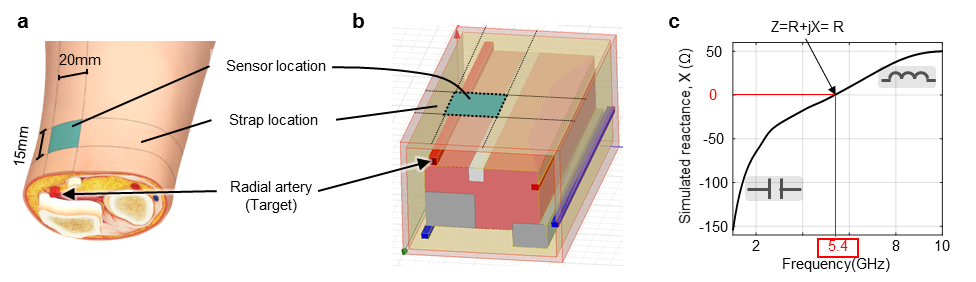
**

**Supporting Figure 3. 3-D electromagnetic simulations for safety standard. a,** the sensor model for simulations. **b–d,** electromagnetic safety simulation results for (**b**) electric field intensity (E-field), (**c**) magnetic field intensity (H-field), and (**d**) specific absorption rate (SAR), respectively.

**
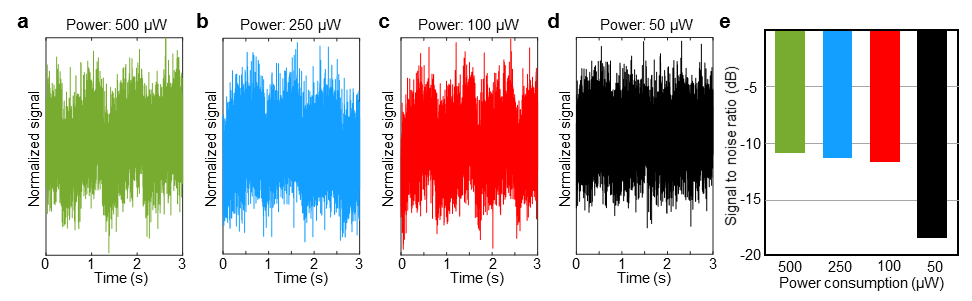
Supporting Figure 4. Optimal power consumption determination. a–d,** experiment results of the proposed sensor using different power consumptions: (**a**) 500, (**b**) 250, (**c**) 100, and (**d**) 50 μW. the sensor is attached on the wrist above the radial artery. **e,** signal to noise ratio comparison of the various power input. Signal to noise ratio is used as the performance evaluation. Too low signal to noise ratio value is observed under 100 μW.

**
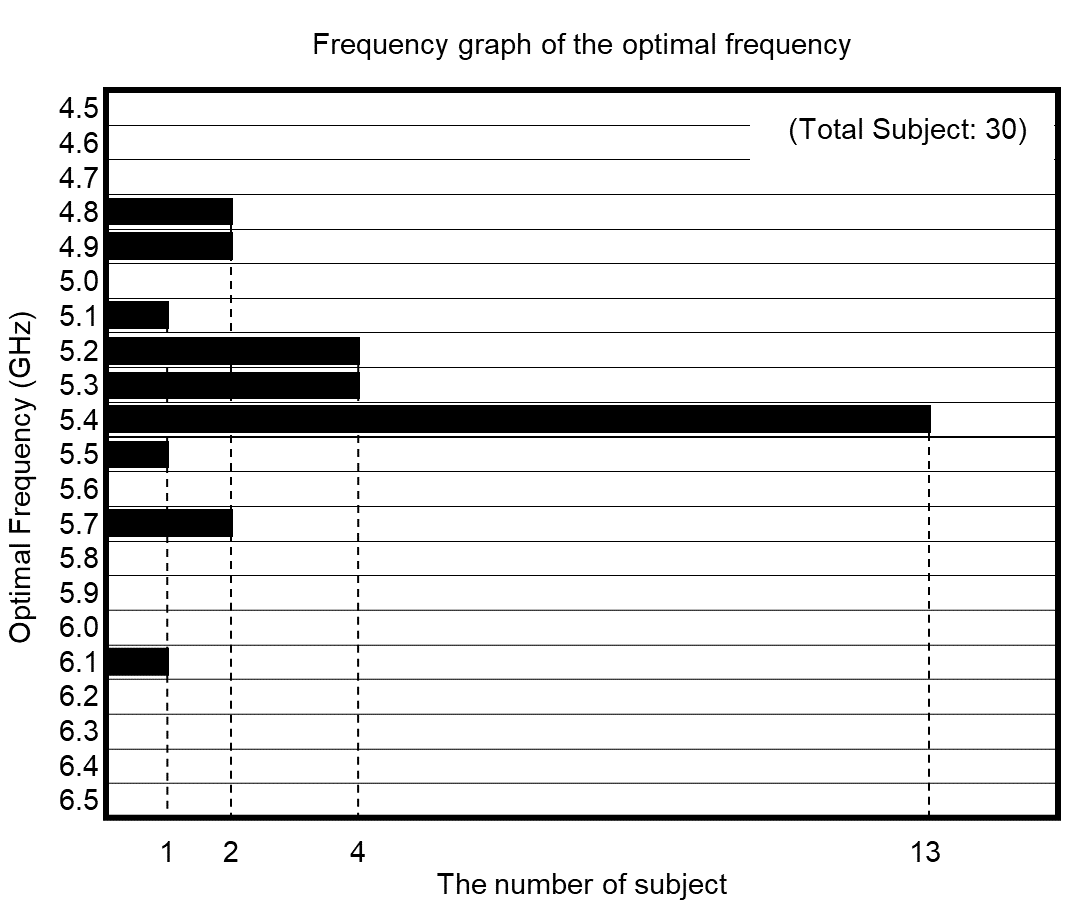
**

**Supporting Figure 5. The frequency graph of the optimal frequency**

**
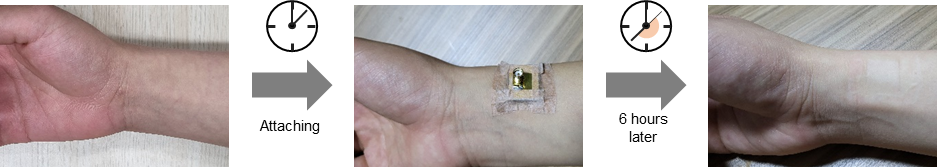
**

**Supporting Figure 6. The pictures of the effect of sensor to human skin.**

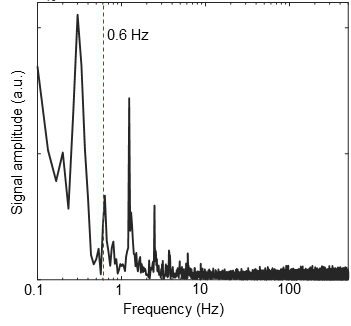
**Supporting Figure 7. Demodulation of respiratory signal.** (a) Raw data for 30 sec. (b) Signal in time-domain after the band-pass filtering. (c) Finally obtained respiratory signal

**Supporting Figure 8. Measured data in the frequency domain**

**Supporting Figure 9. Modulated cardiovascular and respiratory signal**

**Supporting Figure 10. A comparison between demodulated respiration with different cut-off frequency ranges**

**Supporting Figure 11. A comparison between demodulated cardiovascular signal with different cut-off frequency ranges**


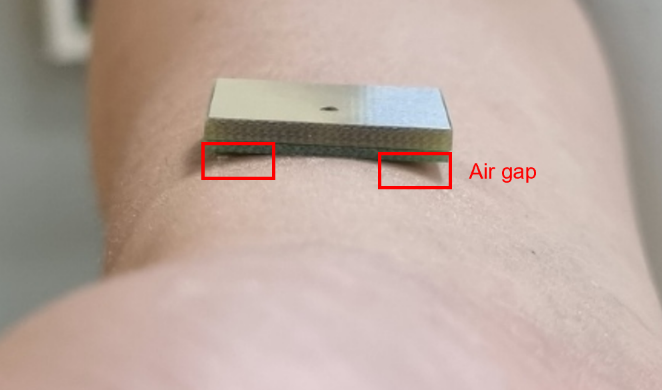
**Supporting Figure 12. The proposed sensor using FR-4 is attached to the skin with a small air gap.**


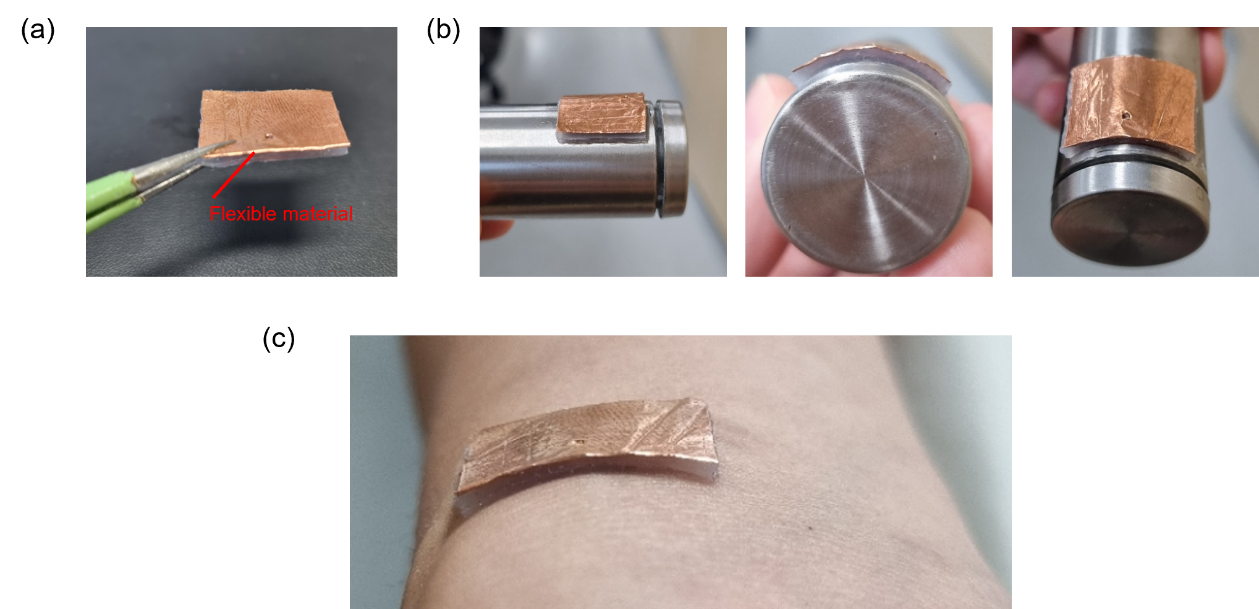
**Supporting Figure 13. Blood pressure monitoring sensor fabricated with a flexible material.** (a) the picture of the fabricated sensor. (b) the flexibility of the sensor is verified. (c) the sensor is attached to the skin without an air gap.

**
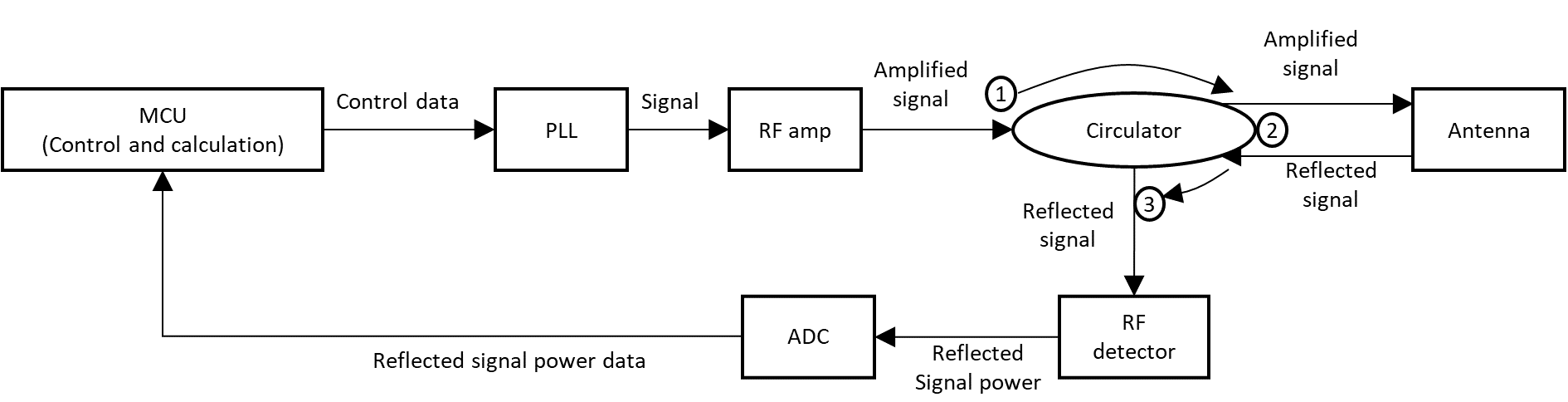
**

**Supporting Figure 14. The expected block diagram of the miniaturized measurement circuit.**


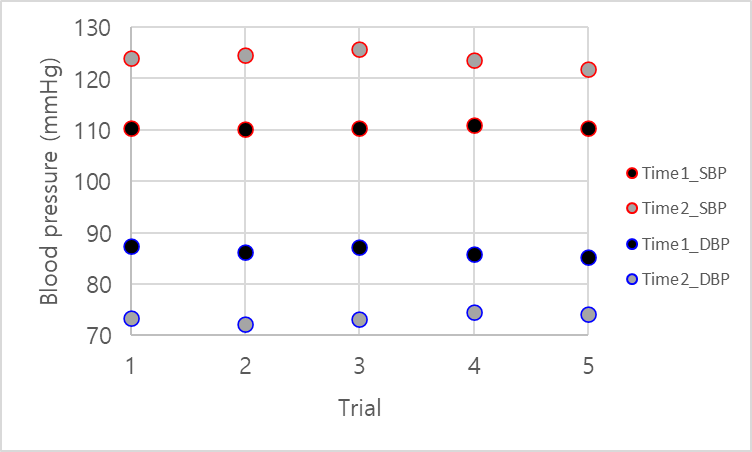


**Supporting Figure 15. Reproducibility check.**


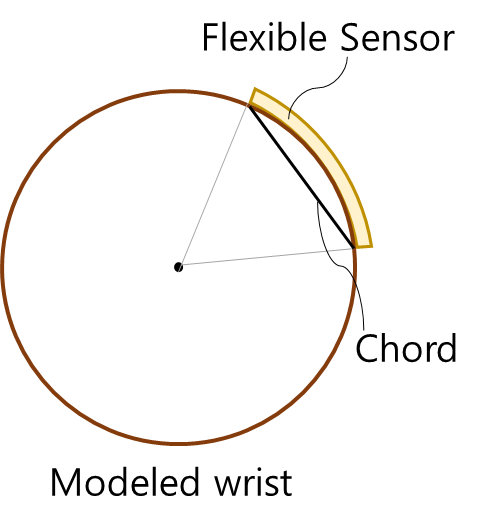


**Supporting Figure 16. Wrist model for curvature of the flexible sensor.**


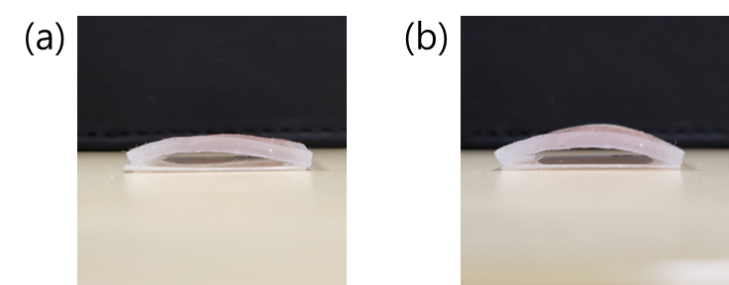
**Supporting Figure 17. Curved sensors based on the wrist curvature of the (a)90^th^ percentile of the males and (b) 10^th^ percentile of females.**


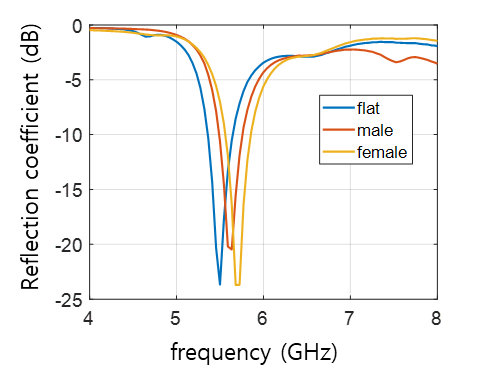
**Supporting Figure 18. Resonant frequencies of the flexible sensors with various mechanical deformations**

**Supporting Figure 19. Blood pressure signal from the flexible sensor.**

**
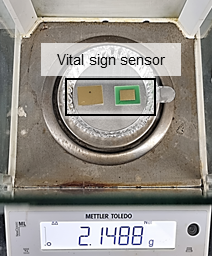
**

**Supporting Figure 20. The fabricated sensor on an electronic scale.** The weight of sensor designed for high usability is only 2.15 g.

**Supporting Tables**

| **Criteria** | **Frequency range (GHz)** | **SAR (W/kg)** |
| --- | --- | --- |
| **Guideline of IEEE** | **2-300** | **10** |
| **The proposed device** | **5.4** | **0.04** |

* SAR: specific absorption rate

**Supporting Table 1. The safety comparison with guideline of Institute of Electrical and Electronics Engineers (IEEE)**

| **Criteria** | **Frequency range**  **(GHz)** | **E-field (V/m)** | **H-field**  **(mA/m)** | **B-field**  **(μT)** | **SAR (W/kg)** |
| --- | --- | --- | --- | --- | --- |
| **Guideline of ICNIRP** | **2-300** | **61** | **160** | **0.20** | **10** |
| **The proposed device** | **5.4** | **50** | **29** | **0.04** | **0.04** |

*** E-field: electric field intensity, H-field: magnetic field intensity, B-field: magnetic flux density, SAR: specific absorption rate**

**Supporting Table 2. The safety comparison with guideline of International Commission of Non-ionizing Radiation Protection (ICNIRP)**

|  | **10^th^ percentile of females** | **90^th^ percentile of males** |
| --- | --- | --- |
| **Radius of curvature (mm)** | **24.6** | **35.0** |
| **Angle (deg)** | **50.3** | **35.4** |
| **Length of chord (mm)** | **20.9** | **21.3** |

**Supporting Table 3. Dimensions of modelling for curvature of the wrist**

**Supporting Video**

Video S1. Vital sign monitoring demonstration
